# Intergenerational education mobility in India: nonlinearity and the Great Gatsby Curve

**DOI:** 10.3389/fsoc.2024.1295550

**Published:** 2024-09-26

**Authors:** P. K. V. Kishan, Abhinav Rajverma

**Affiliations:** ^1^Department of Economics, Institute of Rural Management Anand, Anand, Gujarat, India; ^2^Department of Finance, Institute of Rural Management Anand, Anand, Gujarat, India

**Keywords:** intergenerational education mobility, education research, intergenerational persistence, educational stratification, educational, inequalities, public policy, Great Gatsby Curve

## Abstract

**Introduction:**

Intergenerational education mobility, which reflects the degree to which an individual’s educational attainment is independent of their parents’ education, is essential for promoting equal opportunities in society. In the context of India, where socio-economic disparities are deeply entrenched, understanding the dynamics of intergenerational mobility is particularly crucial.

**Methods:**

This paper examines various aspects of intergenerational education mobility in India using data from the Indian Human Development Survey (IHDS), a nationally representative multi-topic survey. We analyze intergenerational mobility across different age cohorts and investigate the nonlinearities in the transmission of education. Additionally, we explore the impact of educational inequality, economic growth, and public expenditure on education on mobility outcomes.

**Results:**

Our analysis reveals a high degree of intergenerational persistence in education, although this persistence has decreased over time. Employing quantile regressions, we observe significant nonlinearities in the relationship between fathers’ and sons’ educational outcomes across the educational distribution. In particular, we find a widening mobility gap between historically advantaged subgroups (urban populations, upper castes, Hindus) and disadvantaged groups (rural populations, lower castes, Muslims) at the middle and upper quantiles. Moreover, we confirm the “Higher Inequality leading to Lesser Mobility” nexus, supporting the ‘Great Gatsby Curve’ within the Indian context. Macroeconomic factors, such as economic growth and public expenditure on education, are positively correlated with educational mobility, suggesting that these factors play a critical role in enhancing mobility.

**Discussion:**

These findings highlight the importance of targeted policy interventions to reduce educational disparities and promote greater intergenerational mobility. The widening mobility gaps between socio-economic and demographic groups emphasize the need for more equitable resource distribution and educational reforms. Future research should explore the multifaceted aspects of intergenerational mobility, incorporating longitudinal studies and regional analyses to deepen our understanding of the underlying mechanisms.

## 1 Introduction

Intergenerational mobility, the extent to which individuals move beyond their social origins and their parents’ socio-economic status, is a crucial indicator of social equity and opportunity (Chetty et al., 2014; Liana et al., 2016). When studying intergenerational mobility, sociologists prefer using measures based on occupations, while economists are more inclined to employ measures focused on earnings and income levels (Torche, 2015). In contrast, intergenerational education mobility specifically examines the ability of individuals to attain higher educational levels than their parents, wherein education serves as a primary vehicle for social and occupational mobility and a significant mediator of intergenerational reproduction (Blau and Duncan, 1967; Sewell and Hauser, 1975; Hout and DiPrete, 2006). Education not only reproduces socio-economic advantages across generations but also provides the main avenue for mobility by allowing individuals to leave behind the disadvantages of their birth (Hout and DiPrete, 2006; Torche, 2015). In this context, understanding intergenerational education mobility is essential to address broader social inequalities.

Historically, India’s education system has been deeply influenced by hierarchical structures rooted in caste and socio-economic disparities (Drèze and Sen, 2013). Since independence in 1947, the Indian government has implemented various policies and reforms to enhance educational accessibility. Initially, the focus was on establishing a unified educational framework to replace the colonial system (Bhattacharya, 2002). Over the years, National Education Policies (NEPs) have sought to address educational disparities and promote equal opportunities. The NEP of 1968 emphasized a uniform educational structure and regional languages to bridge inequalities (Ministry of Education, 1968). The 1986 NEP further aimed to improve educational quality and access, particularly for marginalized communities, through initiatives like “Operation Blackboard” and expanded scholarship schemes (Ministry of Education, 1986). The Programme of Action in 1992 focused on universal elementary education and decentralized administration (Ministry of Education, 1992).

Affirmative action policies, such as reservations in education and employment for Scheduled Castes (SCs), Scheduled Tribes (STs), and Other Backward Classes (OBCs), have played a crucial role in promoting educational equity (Desai and Kulkarni, 2008). Initiatives like Sarva Shiksha Abhiyan (SSA) in 2001 aimed for universal primary education, significantly increasing enrollment rates and literacy. The literacy rate rose from 18.3% in 1951 to 74.04% in 2011 (Census of India, 2011), and the mean years of schooling increased from 2.4 years in 1950 to 6.7 years in 2021 (World Bank, 2021). However, educational attainment across various population groups remains uneven, and learning outcomes are strikingly low, with significant disparities in basic reading and arithmetic skills among rural students (ASER, 2023). A recent nationwide rural household revealed that as of 2022, only 20.5 percent of grade three students in rural schools could read a grade two textbook, and only 25.9 percent of grade three children could do basic arithmetic. Additionally, only 38.5 percent of grade five students can at least read at the grade two level, and 41.8 percent of grade eight children can divide numbers (ASER, 2023).

Given this backdrop, our study explores various aspects of intergenerational educational mobility in the country. We choose education as our primary lens to study intergenerational mobility as it is less prone to errors than income in terms of measurement. In developing countries like India, data on educational outcomes is more readily available than data on earnings and income, which are typically scarce in household surveys (Azam and Bhatt, 2015). Educational attainment through formal education becomes fixed for individuals by their mid-twenties, thereby mitigating life cycle biases that can affect income measures over time (Haider and Solon, 2006; Black and Devereux, 2011). In this study, we use “years of schooling” to characterize educational outcomes. We acknowledge that a complete understanding of educational inequalities and intergenerational education mobility in India would require an inquiry and evaluation of educational quality and learning outcomes. However, in light of inadequate longitudinal or panel data sources that also track students’ learning achievements and competencies, ‘years of schooling’ remains an important indicator, especially where a substantial proportion of children still do not complete the requisite years of schooling (Jalan and Murgai, 2008).

Prior studies on intergenerational education mobility in India have predominantly concentrated on descriptive measures of intergenerational mobility, such as intergenerational regression coefficients and correlations (Jalan and Murgai, 2008; Maitra and Sharma, 2009; Hnatkovska et al., 2013; Azam and Bhatt, 2015; Emran and Shilpi, 2015; Choudhary and Singh, 2017, 2019). These studies have ably established a baseline understanding of the state of intergenerational educational mobility in India. However, the current literature falls short of capturing the complex mechanisms underlying the transmission of advantages and disadvantages from parents to children or the factors that promote intergenerational education mobility. Incorporating the human capital approach to inequality, Becker and Tomes (1979) establish determinants of intergenerational mobility through their model. Per the model, a child’s future outcomes depend on the degree of inheritability of endowments (of multiple traits including IQ, ability, and reputation), parents’ propensity to invest in their human capital, and a random ‘luck’ component. Further, other factors such as economic growth rate, tax subsidy, public expenditure systems, and discrimination against minorities sometimes have surprising implications on intergenerational transmission of advantage. Literature focusing on the correlates of intergenerational mobility, including macro-level factors such as public spending on education and economic growth, is limited in the developing countries’ context.

Additionally, there is a lack of consensus on how intergenerational mobility varies across the educational distribution of children, leaving the nonlinearities in the persistence of educational attainment underexplored. These gaps highlight the need for a more comprehensive analysis of the intergenerational transmission of educational advantages.

This study aims to fill these gaps by examining intergenerational education mobility in India, focusing on two main aspects. First, we investigate the nonlinearities in the intergenerational transmission of education, exploring how the association between parents’ education and their childrens’ education varies across different levels of the educational spectrum. Second, we analyze the relationship between educational inequality in one generation and intergenerational education mobility in the next, in line with the Great Gatsby Curve. We also assess the role of economic growth and public expenditure on education in influencing mobility. By addressing these aspects, this study contributes to a deeper understanding of intergenerational education mobility in the context of a developing economy, offering valuable insights for policymakers aiming to promote educational equity.

We employ the latest round (2011–2012) of Indian Human Development Survey (IHDS-II) data and utilize the retrospective information provided for the educational attainment of the father (husband) of the male (female) head of the household to prepare a representative dataset consisting of 44,532 adult males (age group 25–64) with paired educational details of their respective fathers. The retrospective information helps to preclude the “co-resident only” sample restriction.

The study’s main findings are: There is concavity in the relationship between fathers’ and sons’ schooling outcomes across the conditional education distribution. Additionally, the mobility gap between an urban citizen and a rural resident, a person belonging to the youngest age cohort vs. one belonging to the oldest cohort, an upper-caste vs. an OBC/SC/ST, a Hindu vs. a Muslim, increasingly widens along the middle and upper quantiles of the educational distribution. Next, we obtain a negative relationship between education inequality in the fathers’ generation and intergenerational mobility in education, thus confirming the ‘Great Gatsby Curve’ phenomenon. Furthermore, economic growth and public spending in education positively correlate with education mobility, lending credence to their respective roles in leveling the playing field.

In the next section, we present a review of the literature. The third section contains a discussion of the methods and data employed in the study. We lay down the results in section four and conclude in the final section.

## 2 Review of literature

### 2.1 Theoretical and empirical evidence on intergenerational mobility

Most of the literature on intergenerational mobility is rooted in Becker and Tomes’ (1979) human capital model. The model specifies that the parents maximize their utility, wherein the utility function spans the next generations and incorporates their children’s future earnings. The model thus demonstrates how parental investments, hinged on the utility function, explain their children’s future outcomes. Based on Becker and Tomes (1979), Solon (1999) presents an interpretation of the intergenerational income correlation via a theoretical model. Solon extends the model in a later study (Solon, 2004) to account for progressive public investment in children’s human capital (public investment in human capital is progressive with reference to parental income). In tune with expectations, the model shows that intergenerational income mobility increases with the increasing progressivity of public investments in human capital. The Solon (2004) model also depicts the theoretical framework intrinsic to the standard empirical procedure of estimating intergenerational persistence wherein the correlation/elasticity between parents’ socio-economic status and that of their adult children is computed. The sign and magnitude of these correlations can help evaluate a society’s success or failure in providing equality of opportunity to children from various family backgrounds based on the rate of transmission of interpersonal equality (Hertz et al., 2008).

Sociology literature provides additional insights into intergenerational education persistence or mobility. Boudon’s (1974) *rational choice theory* posits that class differentials in educational attainment arise through two main effects: primary and secondary. Primary effects refer to the differences in academic performance between children from advantaged and disadvantaged backgrounds, often reflected in standardized test scores and exam results. On the other hand, secondary effects involve the choices children and their parents make throughout the educational journey, such as whether to continue schooling or pursue specific academic tracks. These decisions are influenced by evaluations of the costs and benefits of various educational pathways and the perceived probabilities of success or failure (Boudon, 1974). Building on Boudon’s work, Breen and Goldthorpe (1997) developed the *rational action theory*, further exploring the secondary effects of educational choices. The central premise is that when deciding on educational strategies, families act as subjectively rational agents who assess the costs, benefits, and probabilities of success associated with different educational options (such as whether to stay in school or choose between academic and vocational courses). Their choices are shaped by two key factors—the level of risk aversion and the degree of motivation to pursue an educational pathway that would allow their child to attain at least the same class position as themselves (the status maintenance motive).

Breen and Goldthorpe (1997) argue that families from higher-class backgrounds exhibit a stronger aversion to downward social mobility. They are more willing to incur greater educational costs and accept lower probabilities of success to avoid downward mobility and maintain their current class status across generations. In contrast, families from lower class origins may be more inclined to take risks with educational investments as the potential gains from upward mobility outweigh the costs of remaining in their current class position. However, their risk-taking is bounded by financial constraints. This relative risk aversion and status maintenance motivation lead to differential educational choices that minimize downward mobility for higher classes and maximize expected upward mobility for lower classes within their constraints.

Other theoretical frameworks that have been influential in explaining social inequalities during educational transitions (Dumont et al., 2019), in turn affecting the intergenerational transmission of educational advantage, include Bourdeau and Passeron’s (1977) *social reproduction theory* that argues education systems perpetuate social inequalities by favoring the cultural capital of dominant social groups. Further, the *Wisconsin social psychological model of status attainment* (Sewell et al., 1969; Sewell et al., 1970; Sewell et al., 2004) emphasizes the role of socio-psychological factors, such as parental encouragement, aspirations, and peer influences, in shaping educational outcomes.

Most of the early empirical studies on intergenerational mobility deal with the computation of precise estimates of correlations and elasticities between the socio-economic status of parents and their adult children for either a cross-section of countries (Corak, 2006; Jantti et al., 2006; Hertz et al., 2008; Blanden, 2009) or individual countries—Sweden and US (Björklund and Jäntti, 1997), Germany (Couch and Dunn, 1997), United Kingdom (Dearden et al., 1997), Canada (Corak and Heisz, 1999).

Lately, there has been a shift in favor of investigating the causal mechanisms fundamental to the association between a child’s life chances and her parents’ socio-economic status (Black and Devereux, 2011). The channels have ranged from the predetermined genetic component to an individual’s childhood environment. Using sibling correlation as a measure of intergenerational mobility, Björklund et al. (2010) delineate the effect of shared parental and neighborhood factors on an individual’s IQ and abilities. In view of the “nature vs. nurture” debate, by estimating the standard intergenerational regression models separately for Korean–American adopted children and their non-adopted American siblings, Sacerdote (2007) finds evidence supporting the thesis that genetics and infant endowments matter more than nurture in influencing the educational outcomes of individuals. Adopting the Instrumental Variable (IV) approach, Oreopoulos et al. (2008) use the father’s displacement from work as a source of variation in his income, unrelated to any other characteristics, to find the effect on children’s outcomes. Employing the Canadian Administrative panel, they detect a 9% difference in annual earnings in favor of sons whose respective fathers were not displaced compared to similar sons whose respective fathers experienced employment shock.

The causal estimates obtained by different identification strategies (identical twins, adoptees, IV estimation) and across different countries differ based on systematic differences in identification strategies and the violation of their internal or external validity assumptions. These strategies tend to focus on separate parts of the socio-economic status distribution, i.e., while twins are spread evenly across the status distribution, adopted children generally belong to the higher end of the distribution, and employment shocks, on average, affect those belonging to the lower end of the distribution (Holmlund et al., 2011).

In a further explanation of mechanisms that underlie the intergenerational transmission of educational advantage, thereby explaining social inequalities in society, Dumont et al. (2019) highlight how parents strategically navigate the educational system to optimize their children’s outcomes, emphasizing the rational choices made to enhance educational attainment. Their mixed-method evidence from Germany illustrates how parents influence educational trajectories, reinforcing the importance of secondary effects in educational attainment (Boudon, 1974; Breen and Goldthorpe, 1997). Additionally, patterns of educational choice reflect evaluations made of the costs and benefits of possible alternatives, such as whether to leave school or stay on and whether to take a more academic or vocational course (Breen and Goldthorpe, 1997).

de Werfhorst et al. (2010) provide a comparative perspective on how institutional structures of educational systems contribute to achievement inequality. Their research indicates that the design of educational systems, such as tracking, vocational training, and standardized testing, plays a significant role in shaping the extent of intergenerational education mobility. Systems that offer more comprehensive support and less stratification tend to exhibit higher levels of educational mobility, suggesting that institutional context is crucial in mitigating or exacerbating educational inequalities. Pfeffer’s (2008) empirical findings align with de Werfhorst et al. (2010) in that Pfeffer (2008) highlights the role institutional contexts play in shaping persistent inequality in educational attainment across generations. By examining various educational systems and their impact on intergenerational mobility, Pfeffer (2008) underscores the significance of institutional structures, such as tracking systems, school funding policies, and standardized testing. His findings reveal that educational policies and institutional frameworks profoundly influence the degree of educational mobility and the perpetuation of socio-economic disparities.

In summary, the above studies highlight the multifaceted nature of intergenerational mobility and its patterns within and across societies by considering both economic and sociological perspectives. These studies reveal that understanding intergenerational education mobility requires a comprehensive approach that considers genetic, environmental, and institutional factors, as well as the rational choices made by families within their specific socio-economic contexts. In addition, given that households and families with different resource endowments face diverse challenges and tradeoffs, the effect of a parent’s education on a child’s achievement may differ significantly between those at the lower and upper ends of the educational distribution. In this paper, we explore these nonlinearities in the context of India.

### 2.2 The Indian setting

India’s economic growth since the 1990s has coincided with increasing inequalities in outcomes (Jayadev et al., 2007; Bharti et al., 2024), raising concern about whether it reflects inequalities in societal opportunities. The Indian society is deeply stratified by caste and beset by poor outcomes and low mobility (Das, 2004). Furthermore, as Maitra and Sharma (2009) contend, this lack of mobility excludes many parts of our society from reaping the rewards of the prolific growth levels the country has experienced during the last three decades.

#### 2.2.1 Indian caste system

The caste system in India, a deeply rooted social hierarchy, traditionally divides society into four main varnas: Brahmins (priests and scholars), Kshatriyas (warriors and rulers), Vaishyas (traders), and Shudras (laborers and artisans) (Deshpande, 2010; Goghari and Kusi, 2023). Additionally, there are the Dalits, who were historically marginalized and considered outside the caste system (Deshpande, 2010). This stratification has profoundly influenced social dynamics and access to resources, including education (Deshpande, 2006).

Efforts to promote equality of opportunity, particularly in education, have been significant in modern India. Policies such as reservations in educational institutions and government jobs aim to improve access for three marginalized categories of caste—Scheduled Castes (SCs) (comprising mainly of Dalits), Scheduled Tribes (STs) (comprising of the Adivasis, the Indigenous peoples), and Other Backward Classes (OBCs) (communities identified as below the national average on social or educational indicators (Ramaiah, 1992)) (Deshpande, 2010; Goghari and Kusi, 2023). Despite these measures, disparities persist. Brahmins and other upper castes often have better access to quality education and economic resources, while SCs, STs, and OBCs still face substantial challenges due to socio-economic barriers (Thorat and Neuman, 2012).

#### 2.2.2 Indian educational system

In India, the education system is structured into several stages, each with a specific duration. The foundational stage is pre-primary education, typically spanning 2 years. Next, the primary stage (the first formal stage) covers grades 1–5, spanning 5 years. Next is middle-school education, encompassing grades 6–8 over 3 years. Secondary education follows, comprising grades 9 and 10 and lasting 2 years. The final stage is higher or senior secondary education, which includes grades 11 and 12, taking 2 years to complete. The formal (K–12) schooling from primary to higher secondary education spans 12 years (GOI, 1992). Beyond higher secondary, students may pursue undergraduate (3–4 years), post-graduate (2–3 years), and doctoral degrees.

Some of the significant objectives of Indian educational planning since the country’s independence include ensuring equity in education by gender, caste, and socio-economic groups, as well as reducing regional disparities in education development (Tilak, 2007). However, the Indian schooling system still exhibits significant stratification, effectively dividing students into separate education tracks with divergent educational and career outcomes. This stratification manifests in the coexistence of well-resourced private schools, government-aided private schools, low-fee private schools, and under-resourced government/public schools (Chattopadhay and Roy, 2017). There is considerable variation in education quality across these categories of schools (Kingdon, 2009).

Private schools, often catering to wealthier segments of society, prepare students for post-secondary education through rigorous curricula and access to extensive resources. On the other hand, low-fee private (LFP) schools are primarily characterized by low teacher salaries and benefits (Muralidharan and Kremer, 2008) and limited infrastructure because they charge low fees as they cater to low-income households. Although these LFP schools provide low-quality education, they attract low-income families due to their promise of English as a medium of instruction^1^ (Chattopadhay and Roy, 2017). In contrast, many government schools, particularly those in rural and underserved areas, levy subsidized fees or provide free education but struggle with inadequate infrastructure, insufficient teaching staff, teacher absenteeism, and poor educational outcomes (Mehrotra and Panchamukhi, 2006; Muralidharan and Kremer, 2008; Drèze and Sen, 2013). Drèze and Sen (2013)) further highlight the disparity in educational quality and opportunities between different types of schools, emphasizing that children from marginalized communities often find themselves in underperforming schools that offer limited pathways to higher education.

Despite the Indian constitution’s commitment to providing free and universal primary and middle-grade education (grades 1–8, ages 6–14) by 1960 and subsequent emphasis in the National Educational Policies of 1968 and 1986, effective and efficient resource allocation has hindered this goal (Cheney et al., 2005). Further, per Cheney and co-authors, although primary and middle school education is compulsory, only about 70% of children aged 6–14 attend school on average. For those who do attend, significant disparities exist in access to education, quality, and learning outcomes based on gender, social class, and location. Additionally, dropout rates for children from the poorest households are four times higher than those from the wealthiest (Cheney et al., 2005).

The stratification is further reinforced after the compulsory grade 10 public exams, where students are funneled into distinct educational streams—Science, Commerce, and Arts/Humanities—for their last 2 years (grades 11 and 12) before university (tertiary education). This tracking system, based on students’ performance in these crucial exams, significantly influences their future educational and career opportunities (Cheney et al., 2005; Jain et al., 2022). The Science stream, considered the most prestigious, opens pathways to professional engineering, medicine, and technology courses and is associated with higher earnings than humanities and commerce (Jain et al., 2022). Only those students who study Science as their higher secondary school major are eligible for STEM courses at the tertiary level (Jain et al., 2022) after the compulsory grade 12 public examinations. In contrast, the commerce and arts streams often lead to less lucrative and socially esteemed career options (Cheney et al., 2005).

This early bifurcation exacerbates educational inequality, as mostly students from privileged backgrounds are more likely to access the resources necessary to excel in the highly competitive science stream (Cheney et al., 2005). Beyond higher secondary education, Azam and Blom (2009) provide evidence to show significant gaps in tertiary or higher education enrolment between poor and rich households, rural and urban areas, lower castes and upper castes, and across states. Azam and Blom (2009) argue that lack of information about higher education, low expectations of attending higher education, and inadequate preparation are some of the underlying reasons behind the unequal access to tertiary education.

Additionally, India’s vocational education system, designed to provide alternative career pathways, can be viewed as a ‘dead-end’ track. This perception stems from the limited scope for upward mobility and weak integration with higher education frameworks (Mehrotra, 2014). Students who enter vocational tracks frequently encounter barriers and challenges that preclude them from attaining higher levels of education (Pilz and Regel, 2021), thereby perpetuating socio-economic disparities.

In summary, these systemic divisions in the Indian educational system not only reinforce existing social hierarchies but also limit access to post-secondary education and high-quality employment opportunities for large segments of the population, potentially contributing to low intergenerational education mobility in India.

#### 2.2.3 Intergenerational mobility studies in the Indian context

Jalan and Murgai (2008) employ two rounds of the National Family Health Survey (NFHS) from 1992–93 to 1998–99 to study inequality in educational attainments and its persistence across generations for different population groups in India. Their results reflect significant and consistent improvements in education mobility and decreasing education gaps between various caste groups. Maitra and Sharma’s (2009) investigation of the intergenerational transmission of human capital using data from the Indian Human Development Survey (IHDS-I) in 2004–05 affirms the results obtained by Jalan and Murgai (2008).

On the contrary, Azam and Bhatt (2015) observe a high degree of intergenerational stickiness in educational attainment. Their sample construction design harnessed the retrospective information provided by IHDS-I on the father’s (or husband’s) educational attainment of the head of the household. The final sample circumvented the “co-resident only” son-father pair constraint (encountered in the use of other large sample datasets in the earlier studies). It consisted of son-father matched pairs representative of the adult male population of India. Concurrently, Emran and Shilpi (2015) draw on 1992–93 and 2006 rounds of NFHS and report Sibling Correlation (SC) and Intergenerational Correlation (IGC) for similar age cohorts as other studies. They find strong intergenerational persistence in education unchanged over the time of the study. When accounted for neighborhood fixed effects, geographic location emerged as an essential factor in the measurement of sibling correlation and intergenerational correlation, suggesting the importance of regional factors in promoting intergenerational education mobility.

Focusing on the women’s end of intergenerational mobility in India, the body of work by Choudhary and Singh has investigated upward and downward intergenerational education mobility (Choudhary and Singh, 2017, 2019) and intergenerational occupational mobility (Choudhary and Singh, 2018b) for young females (vis-à-vis their mothers) using transition matrices at the all-India level and also disaggregated by states, analyzed the association between intergenerational educational mobility and the overall health of Indian women (Choudhary and Singh, 2018a), and estimated inequality of opportunity (IOp) among Indian women by considering parental education, caste, religion, and region of birth as circumstance variables (Choudhary et al., 2019). In one of the latest studies, (Asher et al., 2022) employ a new measure of upward mobility that estimates the expected education rank of a child (for both sons and daughters) born to the parents in the bottom half of educational attainment distribution across various castes and religions in India over time.

The studies on intergenerational education mobility in India have differed in terms of the choice of measures and data sources. Although a consensus has not emerged, some studies agree upon improvements in education mobility in India and attribute various reasons to the process, ranging from structural changes following liberalization to positive discrimination policies. However, there is a lack of literature in the Indian context to ascertain the channels underlying the transmission of advantage from one generation to the next. In this paper, we investigate the effect of a few macro-level factors on intergenerational education mobility.

## 3 Materials and methods

Most evaluations on intergenerational mobility are carried out by either assessing variables across a repeated cross-section of the population or by measuring the variables across age cohorts (Bussolo et al., 2019). We commence by conducting an analysis of the trends in intergenerational education mobility by dividing the sample of individuals into the youngest and the oldest 10-year birth cohorts and estimating the following model using ordinary least squares (OLS) regressions:


(1) $$S_{i}=\beta_{0}+\beta_{1}F_{i}+X_{i}^{\prime }\;\theta +\epsilon_{i}$$

where $S_{i}$ denotes the number of years of schooling of the *i*th son, $F_{i}$ (the circumstance variable) is *i*th father’s completed years of schooling, ${X_{i}}^{\prime }$ is a vector of control variables that includes dummies for a son’s caste, religion, and state, and $\epsilon_{i}$ is the i.i.d. error. $\beta_{1}$ is the primary variable of interest and is termed the Intergenerational Regression Coefficient (IGRC). $\beta_{1}$ captures the sensitivity of the expected educational outcome of the sons to unit changes in the educational attainment of the fathers. It conveys how strongly past circumstances affect the educational attainment of the son and, in turn, his life chances.

Equation 1 can be further estimated by adopting either the co-resident household approach or the two-sample instrumental variables approach (Shariq, 2019). The three major sample surveys in India—National Sample Survey Organization’s thick sample Consumption Expenditure survey and Employment-Unemployment Survey, National Family Health Survey, and Indian Human Development Survey—amply facilitate the co-resident household approach. However, considering only co-resident son-father pairs might generate attenuation bias as cohabitation might be systematically linked to decisions regarding human capital investments in a household. Moreover, as Motiram and Singh (2012) posit, we would be missing out on single-member households, two-member households consisting of husband and wife, and nuclear families (husband, wife, and children), which would by itself lead to a substantial loss in observations. Such a truncation in the number of observations leads to a downward bias in IGRC. As established by Emran et al. (2018), the bias is inversely proportional to the extent of co-residency rates observed in the data.

We exclude females in this analysis for the following reasons. One, households with women as heads are very few (2.95% of all cases). Even for such households, education data is provided for their husbands. Hence, the unique feature of the IHDS data cannot be utilized to create a representative sample of such pairings for daughter-father, daughter-mother, or son-mother pairings. Two, given the ubiquitous family structure in India, adult females reside in either nuclear households or joint families along with their respective husbands and kin belonging to the husband’s side. Hence, the requisite pairing information is unavailable for a purported representative sample, even if we only consider the co-residency condition.

To carry out the analyses, we employ the second round of the Indian Human Development Survey (IHDS-II) conducted in 2011–12. IHDS is a collaborative project between the National Council of Applied Economic Research (NCAER) and the University of Maryland. The survey is nationally representative and covers 42,152 households in 1,420 villages and 1,042 urban neighborhoods across India. It includes household information on education, health, employment, economic status, social capital, fertility, etc.

We prepare a dataset aligned with Azam and Bhatt’s (2015) approach. The dataset is unique because, besides matching father-son data based on the “Relationship to head of household” field in the household questionnaire that links the co-resident pairs, we also use the retrospective question (1.18c on page 3 of the Income and Social Capital Questionnaire) on the household head’s educational attainment. The survey question enquires about the educational attainment of the father/husband of the head of the household. The final sample consists of 44,532 observations of individuals (or sons) aged between 25 and 64 (as of 2012) with matched information on their respective father’s educational attainment. Refer to Table 1 for a summary and description of all the variables in this article.

**Table 1:** Description and sources of main variables.

Variable	Description	Source
Son’s YoS	Son’s educational attainment in terms of completed years of schooling	IHDS-II
Child’s YoS	Son’s or Daughter’s educational attainment in terms of completed years of schooling	IHDS-II
Father’s YoS	Father’s educational attainment in terms of completed years of schooling	Own calculations using IHDS-II
Mother’s YoS	Mother’s educational attainment in terms of completed years of schooling	Own calculations using IHDS-II
Age	Son’s age in years	IHDS-II
$G_{s}$	Education Gini Coefficient in Father’s generation in gtate *s*	Own calculations using IHDS-II
$E_{s}$	Per capita expenditure on education as a proportion of Gross State Domestic Product (GSDP) per capita in state *s*	Own calculations using the CMIE States of India Statistical Compendium
$R_{s}$	Year-on-year per capita GSDP growth in state *s*	Own calculations using EPWRF India Time Series
Female	Dummy = 1 if the individual is a female	Own calculations using IHDS-II
Year of birth	Year of birth of the son	IHDS-II

We operationalize educational attainment as the number of years of schooling. Besides the number of years of schooling or education, IHDS-II reports a variable, ‘EDUC7’, that encompasses completed years of education across seven categories (excluding the category, ‘None,’ i.e., zero years of schooling). The distribution of these seven education categories in our sample is presented in Table 2.

**Table 2:** Distribution of various categories of education per IHDS-II in the sample.

Categories of education	No. of completed years	N	Percent	Cumulative percent
None	0	8,924	20.04	20.04
Grades 1–4	3	3,733	8.38	28.42
Primary	5	3,605	8.1	36.52
Grades 6–9	8	11,893	26.71	63.22
Secondary	10	6,683	15.01	78.23
Higher secondary	12	4,388	9.85	88.08
Graduate	15	3,489	7.83	95.92
Some post-graduate	16	1,817	4.08	100
Total		44,532	100	

Authors’ calculations.

Although educational credentials such as obtaining a higher secondary school qualification or a college degree are crucial milestones associated with greater economic returns compared to the same number of years of schooling without the qualification due to the signaling function of education (sheepskin effect) (Hungerford and Solon, 1987), we focus on years of schooling or education in our analyses. This approach aligns with current literature on intergenerational educational mobility, which predominantly employs empirical models that utilize this operationalization to estimate linear mobility equations, yielding IGRCs and IGCs (Hertz et al., 2008; Neidhöfer et al., 2018; Torche, 2021; Ahsan et al., 2022). Educational attainment can also be represented by educational categories or credentials. In such cases, mobility measures are derived from transition matrices that cross-classify the educational categories of parents and children (Torche, 2021). However, transition matrix-based intergenerational educational mobility metrics are beyond the scope of our study.

Per the convention in literature (Azam and Bhatt, 2015; Neidhöfer et al., 2018; Ahsan et al., 2022), apart from IGRC, we also report Intergenerational Correlation (IGC). IGC is a standardized measure of intergenerational persistence that removes the cross-sectional variability in educational attainment in successive generations. Equation 2 operationalizes it–


(2) $$IGC=\beta_{1}\left(\frac{\sigma_{F}}{\sigma_{C}}\right)$$

where $\sigma_{F}$ and $\sigma_{C}$ are the standard deviations of educational attainment of the father’s and son’s generation, respectively.

## 4 Results and discussion

The summary statistics for the estimation sample are presented in Table 3, which includes data on the educational attainments of 44,532 males aged 25–64 and their fathers. These age bounds ensure the inclusion of sons who have completed schooling and prevent selection bias due to varying survival rates among different family backgrounds (Behrman et al., 2001).

**Table 3:** Summary statistics for the overall sample and other groupings.

Variable	Observations	Mean	Std. Dev.	Min	Max
Overall					
Son’s YoS	44,532	7.331	4.942	0	16
Father’s YoS	44,532	3.422	4.362	0	16
Rural India					
Son’s YoS	28,138	6.306	4.791	0	16
Father’s YoS	28,138	2.487	3.723	0	16
Urban India					
Son’s YoS	16,394	9.092	4.695	0	16
Father’s YoS	16,394	5.027	4.880	0	16
Brahmins and other upper castes					
Son’s YoS	13,124	9.117	4.755	0	16
Father’s YoS	13,124	5.071	4.891	0	16
Other Backward Castes (OBCs)					
Son’s YoS	17,981	7.084	4.743	0	16
Father’s YoS	17,981	3.150	4.081	0	16
SCs and STs					
Son’s YoS	12,702	5.835	4.842	0	16
Father’s YoS	12,702	2.094	3.551	0	16
Hindu					
Son’s YoS	36,369	7.474	4.930	0	16
Father’s YoS	36,369	3.464	4.383	0	16
Muslim					
Son’s YoS	5,264	5.910	4.900	0	16
Father’s YoS	5,264	2.787	4.023	0	16
Others (Christians, Sikhs, Jains, etc.)					
Son’s YoS	2,899	8.124	4.709	0	16
Father’s YoS	2,899	4.046	4.553	0	16

Son’s YoS—Years of schooling of the sons; Father’s YoS—Years of schooling of the fathers; Rural/Urban classification is as per the 2011 census.

Our findings reveal that sons have higher mean educational attainment than their fathers across all groupings. Educational attainment is higher in urban India than in rural areas for both generations. Brahmins and other upper castes are significantly more educated than lower castes. Among religious groups, Muslims have the lowest educational attainment, while Christians, Sikhs, Jains, and others fare better than Hindus. We also present the mean educational attainment for fathers’ and sons’ generations for successive age cohorts in Appendix Table A1 and rate increases in schooling attainments *per annum* for various subgroups in Appendix Table A2 in Appendix A.

Table 4 presents the OLS estimation results for the overall sample. For the base specification, the estimated IGRC is 0.588 (Table 4), indicating a strong dependency of a son’s educational attainment on his father’s status.

**Table 4:** Intergenerational regression coefficients (all India) (dependent variable—Son’s YoS).

	(1)	(2)	(3)	(4)
Father’s YoS	0.588***	0.544***	0.534***	0.522***
	(0.004)	(0.005)	(0.004)	(0.005)
Constant	5.318***	7.131***	7.325***	8.140***
	(0.027)	(0.083)	(0.162)	(0.165)
Caste dummies	No	Yes	Yes	Yes
State dummies	No	No	Yes	Yes
Religion dummies	No	No	No	Yes
*N*	44,532	44,411	44,411	44,411
Adj. R-sq.	0.270	0.287	0.306	0.316
Correlation (IGC)	0.519	0.479	0.470	0.460

Standard errors clustered at household level in parentheses; *p < 0.05, **p < 0.01, ***p < 0.001.

Next, we control for factors such as caste, state, and religion, which are known to influence schooling achievements (Hickey and Stratton, 2007; Borooah and Iyer, 2005; Asadullah and Yalonetzky, 2012). Once the control variables are accounted for, the degree of persistence decreases, emphasizing the significant role of these factors in educational inequality. The Wald test confirms the equality of the coefficients on fathers’ educational attainment across the specifications in Table 4.

### 4.1 Intergenerational education mobility across cohorts

Caste and religion significantly impact socio-economic outcomes and status in India (Thorat and Neuman, 2012). Hence, we estimate IGRC for different caste and religious groups by age cohorts (25–34 and 55–64) to understand its evolution. These estimates are displayed in Tables 5, 6.

**Table 5:** Cohort trends in intergenerational regression coefficient by caste (dependent variable—Son’s YoS).

	(1)	(2)	(3)	(4)	(5)	(6)
	Brahmins and other UCs		OBCs		SCs & STs	
	25–34	55–64	25–34	55–64	25–34	55–64
Father’s YoS	0.422***	0.596***	0.449***	0.601***	0.447***	0.670***
	(0.013)	(0.019)	(0.012)	(0.022)	(0.015)	(0.038)
Constant	9.190***	6.870***	8.511***	4.372***	7.549***	5.134***
	(0.357)	(0.450)	(0.612)	(0.691)	(0.431)	(1.146)
State dummies	Yes	Yes	Yes	Yes	Yes	Yes
Religion dummies	Yes	Yes	Yes	Yes	Yes	Yes
*N*	4,042	2,217	5,919	2,931	4,313	1,882
Adj. R-sq.	0.337	0.359	0.271	0.233	0.228	0.237
Correlation (IGC)	0.483	0.517	0.445	0.420	0.399	0.396

Standard errors clustered at household level in parentheses; *p < 0.05, **p < 0.01, ***p < 0.001.

**Table 6:** Cohort trends in intergenerational regression coefficient by religion (dependent variable—Son’s YoS).

	(7)	(8)	(9)	(10)
	Hindu		Muslim	
	25–34	55–64	25–34	55–64
Father’s YoS	0.426***	0.599***	0.533***	0.650***
	(0.008)	(0.015)	(0.024)	(0.045)
Constant	8.595***	7.592***	7.159***	3.338***
	(0.326)	(0.492)	(0.404)	(0.498)
Caste dummies	Yes	Yes	Yes	Yes
State dummies	Yes	Yes	Yes	Yes
*N*	11,700	5,858	1,899	771
Adj. R-sq.	0.292	0.351	0.335	0.277
Correlation (IGC)	0.440	0.441	0.472	0.476

Standard errors clustered at household level in parentheses; *p < 0.05, **p < 0.01, ***p < 0.001.

Within all categorizations in Tables 5, 6, we find that all groups have experienced improved educational mobility across generations since independence, though at different rates. SCs and STs show faster improvement than upper castes and OBCs, with a 33.28% decline in IGRC (which indicates increased education mobility) over 30 years compared to 25.29% for OBCs and 29.19% for upper castes (Table 5). Our findings are consistent with Hnatkovska et al. (2013) and Jalan and Murgai (2008).

Among religious groups, Hindus exhibit greater educational mobility than Muslims, with a 28.88% decrease in IGRC over 30 years compared to 18% for Muslims (Table 6). The IGRC for Muslims in the 25–34 cohort is higher than the overall sample’s IGRC in the same cohort. Their educational outcomes continue to be majorly hindered by their previous generations.

While the IGRC estimates indicate a significant improvement in relative educational mobility for the youngest age cohort (25–34) compared to the oldest age cohort (55–64) across the overall sample and subgroups, the IGC estimates in Tables 5, 6 do not reflect this increase. These findings align with Hertz et al. (2008) and Neidhöfer et al. (2018). Ahsan et al. (2022) examine the discrepancies between IGRC and IGC estimates, noting that these metrics can lead to different conclusions. They attribute these differences to the elasticity of IGC with respect to IGRC, which is consistently less than 1, resulting in lower variation in IGC as a measure. Additionally, Ahsan et al. (2022) argue that IGC estimates are less responsive to policy changes compared to IGRC and may not accurately capture the impact of such changes on relative educational mobility within a country.

Lastly, we include mothers and daughters in the estimation sample and consider all sons and daughters aged 11–64 who are no longer enrolled in school and are co-residents with their respective fathers and mothers in one of the models. In the rest of the specifications for the overall sample and sub-samples, we include all individuals (sons and daughters) aged 25–64 who are co-residents with their respective parents. The results are presented in Appendix B (Appendix Table B1).

### 4.2 Nonlinearities

The standard intergenerational education persistence model assumes a linear relationship between sons’ and fathers’ educational attainments. However, several studies have shown, theoretically and empirically, that the relationship could be nonlinear across the educational distribution given credit market imperfections, differences in intra-family altruism, the indivisibility of investment in human capital, and neighborhood effects (Becker and Tomes, 1986, 1979; Galor and Zeira, 1993; Grawe, 2004; Jantti et al., 2006; Bratsberg et al., 2007). Ben-Halima (2012) reasons intergenerational persistence is high at the lower end of the educational distribution due to under-education and poverty traps. As for high intergenerational persistence at the other end of the spectrum, he argues that highly placed families pass on the advantage to the next generation.

To empirically assess the differences in the association between a father’s education and his son’s education across the distribution of the sons’ educational attainments, we employ quantile regression. Equation 3 is estimated for the overall sample and subsamples–


(3) $$Q_{\theta }\left(S_{i}|F_{i}\right)=\beta_{0}+\beta_{\theta }F_{i}+\left(\mathrm{cohort effects}\right)+\in_{i}$$

where $Q_{\theta }\left(S_{i}{\mathrm{|F}}_{i}\right)$ represents θth centile of the distribution of the sons’ educational attainment conditional on the fathers’ years of schooling. The conditional quantiles refer to the son’s years of schooling ranking generated by unobserved characteristics (ability, motivation, genetics, etc.), controlling for the effect that comes from the observables or rather conditional on the observables (Alejo et al., 2021), in this case, father’s years of schooling. The 0.10, 0.20, 0.50 (or median), 0.75, 0.90, and 0.95 quantiles listed in Tables 7, 8 broadly correspond to 0 years (no education), 5 years (completed primary education), 8 years (completed middle-school education), 10 years (completed secondary-school education), 12 years (completed higher secondary-school education), and 15 years (completed some tertiary education) of schooling, respectively, for the sons’ educational distribution in our sample per the mid-rank method (Ahsan et al., 2022).

**Table 7:** Intergenerational regression coefficients across the distribution of sons’ years of schooling (dependent variable—Son’s YoS).

	(1)	(2)	(3)	(4)	(5)	(6)	(7)
	Quantile (0.10)	Quantile (0.20)	Quantile (Median)	Quantile (0.60)	Quantile (0.75)	Quantile (0.90)	Quantile (0.95)
All India							
Father’s YoS	0.667***	0.900***	0.600***	0.500***	0.467***	0.375***	0.200***
Constant	0	0	5***	7***	9***	12***	14***
*N*	44,532						
Rural sample							
Father’s YoS	0.600***	0.800***	0.556***	0.500***	0.400***	0.375***	0.375***
Constant	0	0	5***	6.500***	8.182***	10***	12***
*N*	28,138						
Urban sample							
Father’s YoS	0.750***	0.800***	0.500***	0.467***	0.400***	0.223***	0.091***
Constant	0	0.714***	7***	8***	10***	13***	15***
*N*	16,394						
Age cohort: 25–34							
Father’s YoS	0.714***	0.800***	0.455***	0.438***	0.467***	0.333***	0.111***
Constant	0	1***	7***	8***	9***	12***	14.78***
*N*	14,529						
Age cohort: 55–64							
Father’s YoS	0.667***	0.938***	0.733***	0.700***	0.533***	0.600***	0.500***
Constant	0	0	4***	5***	8***	10***	12***
*N*	7,138						

*p < 0.05, **p < 0.01, ***p < 0.001; We check if the use of quantile regression is justified by employing Breusch-Pagan/Cook-Weisberg test for heteroscedasticity. H_0_: Variance of the error terms is constant. For the Overall Sample, chi2(1) = 2394.11; Rural Sample, chi2(1) = 529.25; Urban Sample, chi2(1) = 915.14; Age Cohort: 25–34, chi2(1) = 563.28; Age Cohort: 55–64, chi2(1) = 76.29. In all cases, Prob > chi2 = 0.0000. Hence, we reject the Null. The use of Quantile Regression is justified.

**Table 8:** Intergenerational regression coefficients across the distribution of sons’ years of schooling (dependent variable—Son’s YoS).

	(1)	(2)	(3)	(4)	(5)	(6)	(7)
	Quantile (0.10)	Quantile (0.20)	Quantile (Median)	Quantile (0.60)	Quantile (0.75)	Quantile (0.90)	Quantile (0.95)
Brahmins and other upper castes							
Father’s YoS	0.75***	0.833***	0.500***	0.467***	0.400***	0.300***	0.100***
Constant	0	1.667***	7.5***	8.267***	10***	12.6***	15***
*N*	13,124						
Other backward castes							
Father’s YoS	0.667***	0.833***	0.500***	0.428***	0.416***	0.375***	0.25***
Constant	0	1***	7***	8***	9.334***	12***	13.75***
*N*	17,981						
SCs & STs							
Father’s YoS	0.555***	0.800***	0.600***	0.500***	0.400***	0.400***	0.400***
Constant	0	0.8***	6***	7.5***	9***	12***	13***
*N*	12,702						
Hindus							
Father’s YoS	0.700***	0.900***	0.555***	0.500***	0.461***	0.375***	0.167***
Constant	0	0.8***	6.667***	8***	9.461***	12***	14.334***
*N*	36,369						
Muslims							
Father’s YoS	0.500***	0.777***	0.667***	0.600***	0.500***	0.500***	0.416***
Constant	0	0	5***	6.4***	8.5***	10.5***	12.25***
*N*	5,910						

*p < 0.05, **p < 0.01, ***p < 0.001.

It is clear from Tables 7, 8 that the association between a father’s education and his son’s schooling is not linear across the sons’ schooling attainment distribution as IGRCs estimated at different conditional centiles of the distribution are not equal. Apropos of the regressions for each subsample, we observe a similar general trend. If we exclude sons with zero educational attainments and thus restrict the sample to between the 20th and 95th centile of son’s educational distribution, intergenerational mobility in education (= 1 – IGRC) displays an increasing trend. However, in some cases, the increase is non-monotonic. For the overall sample, mobility stands at a value of 0.1 at the 20th percentile. It then maintains an upward trend along the rest of the distribution, reaching an (almost) peak value of 0.8 at the 95th percentile (Table 7). This means that the sons with the highest educational attainment are the ones least bound by their circumstances (conditional on their background). Even for the rest of the subsamples (rural, urban, Hindus, Muslims, etc.), this holds, albeit to different extents.

Rural inhabitants are often impeded by the lack of economic and educational opportunities compared to their urban counterparts. As evident in the second and third panels of Table 7, urban areas promote greater education mobility than rural regions. Finally, from the bottom two panels of Table 7, we can safely contend that there has been a marked improvement in educational mobility over the years at almost all points of the education distribution. We also note that the mobility gap between an urban citizen and a rural resident, a person belonging to the youngest age cohort vs. one belonging to the oldest cohort, an upper-caste vs. an OBC/SC/ST, a Hindu vs. a Muslim, increasingly widens along the middle and upper quantiles of the educational distribution (Tables 7, 8). Attributing specific causes behind the source of such differences in mobility rates across various subsamples of the population is beyond the scope of this paper. Nonetheless, in the next section, we shall attempt to shed some light on factors possibly intrinsic to the intergenerational education relationship.

The quantile regression results are comparable to those estimated in Eide and Showalter (1999) (for the USA) and Grawe (2001) (for the USA, Canada, Malaysia, Nepal, and Peru). The results underscore that a son’s background characterized by his father’s educational outcome is the more important explanatory variable for the son’s life chances at the bottom of the son’s conditional education distribution than at the top. Such a “fanning in” pattern of intergenerational association suggests that the dispersion in sons’ educational attainment is wider at lower compared to higher levels of fathers’ schooling distribution. It means a higher probability of sons of highly educated fathers staying homogenously well-educated than the likelihood of sons of less-educated fathers staying homogenously less educated (Torche, 2013).

### 4.3 The Great Gatsby Curve and other channels

The Great Gatsby Curve (GGC) displays a positive relationship between economic inequality in one generation and intergenerational income persistence in the next generation for countries worldwide (Krueger, 2012; Corak, 2013). The curve implies that the persistence in the circumstances handed over by parents to their children depends on the economic inequality prevalent in the said region during the parents’ time. We attempt to see if that indeed is true in the case of education in India. As education is one of the main channels of transmission of income (dis)advantage from parents to children, we estimate the relationship between education inequality experienced by a son while growing up (i.e., education inequality in the father’s generation) and intergenerational education mobility as an adult. Subsequently, we examine the effect of public expenditure on education and economic growth during a son’s childhood on the persistence of educational outcomes. We shall account for cross-state heterogeneities and consider state-level variables.

In most cases, education materializes early on in one’s life. The internal circumstances and the external environment experienced by individuals while growing up shape their outcomes and life chances (Becker and Tomes, 1979; Bourdeau and Passeron, 1977). Suppose inequality in human capital levels among families is high for a given generation. In that case, the subsequent investment inequality in children’s education, directly and indirectly, conserves the status quo and impedes mobility. However, the countervailing forces of education spending by the government (Mayer and Lopoo, 2008; Aizer, 2014) and economic growth (Maoz and Moav, 1999; Hassler et al., 2000) work toward neutralizing the advantage due to better family background and further intergenerational mobility.

Going further, we consider children in the age group 6–18 as differences in mobility rates between two populations are induced by factors that affect individuals in their formative years (Chetty et al., 2014). Given the IHDS-II data, we examine adult sons (aged 25 and above as of 2011) and hence operate with the cohort born during 1974–86. Consequently, we account for state-level variables of *per capita expenditure on education as a proportion of Gross State Domestic Product (GSDP) per capita* and *year-on-year per capita GSDP growth* for 1992–93. To allow for transitory shocks and measurement errors, we average the two variables over 5 years (1990–91 to 1994–95) in place of a single value for the benchmark year 1992–93. Information on the education expenditure variable is obtained from the *CMIE States of India* Statistical Compendium. For GSDP growth rates, we refer *to EPWRF India Time Series* economic indicators. Finally, the Gini of the educational attainment of fathers of sons in the birth cohort 1974–1986 is constructed to denote education inequality in the fathers’ generation. These state-level variables are slow-moving, i.e., they remain relatively stable across time. Thus, following Chetty et al. (2014), we estimate cross-sectional relationships rather than employing panel data methods.

**Figure 1:**
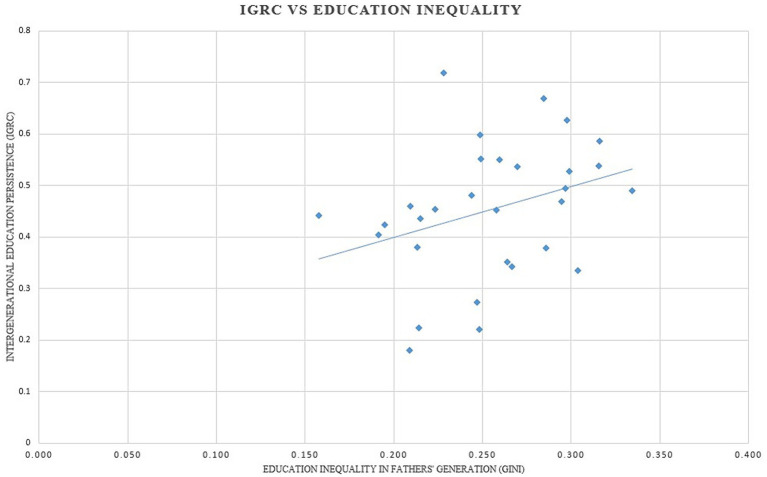
Intergenerational regression coefficient vs. education Gini (Author’s analysis).

Figure 1 plots the relationship between education inequality in fathers’ generation and IGRC for the birth cohort (1974–1986) for various Indian states. The Gini coefficients and IGRCs for respective states are presented in Appendix Table C1 (Appendix C). The cross-state relationship between the variables of interest in Figure 1 corroborates the Great Gatsby Curve connection for education in India. In a state where education inequality is high during the father’s time, a son’s educational attainment and, in turn, his life chances are dictated by his father’s educational status. Hence, in such a state, on average, the son of a relatively less educated father will find it difficult to climb the ladder of progress.

Next, we empirically test the hypothesis of a positive relationship between inequality and intergenerational persistence. We also assess the effect of public expenditure on education and economic growth while a child grows up on his opportunity to move beyond his fathers’ status. Equation 4, based on Neidhöfer (2019) is employed and estimated using OLS regressions:


(4) $$\begin{array}{l} S_{is}=\beta_{0}+\beta_{1}F_{is}+\gamma_{1}*F_{is}*G_{s}+\delta_{1}G_{s}+\gamma_{2}*F_{is}*\\ \;\;\;\;E_{s}+\delta_{2}E_{s}+\gamma_{3}*F_{is}*R_{s}+\delta_{3}R_{s}+\theta_{s}+\in_{is} \end{array}$$

Where the subscript *s* denotes son *i*’s state of residence, $G_{s}$ depicts the education Gini coefficient in fathers’ generation, $E_{s}$ indicates the state government’s expenditure on human capital, $R_{s}$ signifies economic growth, and $\theta_{s}$ encapsulates the state fixed effects. $\gamma_{1}$ , $\gamma_{2}$ , and $\gamma_{3}$ are the coefficients of interest. $\epsilon_{is}$ is the i.i.d. error.

In Table 9, the IGRCs are reported in the top row. The coefficients of the interaction between fathers’ education outcomes and the channels under consideration are presented in rows two to five. There are three main findings. First, we obtain a confirmation of a positive relationship between education inequality and intergenerational education persistence. Evidently, in India’s case, inequality majorly subjects a son’s life chances to depend on his background and lessens the role of hard work. It means that a son of an educationally advantaged father has access to better schools, an opportunity to study further, and better networks than his counterpart with a less educated father. Unless the less educated father can access credit against his son’s potential and invest in the son’s human capital, the circumstantial disadvantage continues onto the next generation, thereby stifling the equality of opportunity. Per Corak (2013), the Great Gatsby Curve phenomenon is also fuelled by increasing returns to education for the highly educated.

**Table 9:** The Great Gatsby Curve and other channels (dependent variable—Son’s YoS).

	(1)	(2)	(3)	(4)	(5)	(6)	(7)	(8)
Father’s YoS	0.498***	0.487***	0.322***	0.282***	0.269***	0.269***	1.385***	2.206***
	(0.006)	(0.006)	(0.045)	(0.047)	(0.053)	(0.054)	(0.150)	(0.213)
*GGC_int*			0.658***	0.777***	1.004***	1.037***	0.289	0.196
			(0.171)	(0.179)	(0.206)	(0.214)	(0.201)	(0.203)
*channel1a_int*					−0.014***	−0.015***		
					(0.003)	(0.004)		
*channel1b_int*							−0.12***	−0.21***
							(0.016)	(0.022)
*channel2_int*						−0.0027		−0.04***
						(0.007)		(0.008)
State fixed effects	No	Yes	No	Yes	Yes	Yes	Yes	Yes
*N*	18,934	18,934	18,934	18,934	18,323	18,286	18,323	18,286
Adj. R-sq	0.251	0.278	0.253	0.278	0.280	0.279	0.281	0.281

Standard errors clustered at household level in parentheses; *p < 0.05, **p < 0.01, ***p < 0.001. We control for the son’s age (Age) in all regressions and report only the pertinent coefficients. Other control variables include $G_{s}$ , $E_{s}$ , and $R_{s}$ . GGC_int is the slope coefficient of the interaction between the Gini of Educational attainment in the fathers’ generation and IGRC. channel1a_int and channel1b_int are the slope coefficients of the interaction between economic growth and IGRC. In channel1a_int, economic growth is—year-on-year per capita GSDP growth (Average from 1990–91 to 1994–95) (in %). In channel1b_int, the definition is—natural log of GSDP per Capita at Constant Prices (1980–81 Series) (Average from 1990–91 to 1994–95); channel2_int is the slope coefficient of the interaction between Government expenditure on education and IGRC. The definition of Government expenditure on education is—Per Capita Expenditure on Education, sports, art & culture as a proportion of GSDP per Capita (Average from 1990–91 to 1994–95) (in %).

Secondly, the negative and statistically significant interaction effect of economic growth with fathers’ education on son’s education points toward a positive relationship between economic growth and intergenerational mobility. This result conforms with the economic models proposed by Maoz and Moav (1999) and Hassler et al. (2000), where growth and mobility reinforce each other. Finally, upholding the empirical findings in Mayer and Lopoo (2008), Blanden (2009), and Aizer (2014), we find a positive effect of public investment in education in reducing the association between a son’s educational achievement and his father’s status. However, the result is not always statistically significant. Higher government spending on education may not always translate into better equality of opportunity. In this regard, Corak (2013) emphasizes the importance of a progressive public spending regime directed toward making quality primary and secondary education more accessible than supplementing resources in higher levels of education accessible to only a few.

## 5 Summary, conclusions, and scope for future research

This paper investigates the role of circumstances in shaping an individual’s life chances in India. While an individual’s (son’s) circumstances are proxied by his father’s education, his life chances are assumed to depend on his educational outcomes. We explore the nonlinear relationship between the educational outcomes of successive generations for various cohorts and regions by employing quantile regressions. We also analyze the role of specific channels—education inequality in the fathers’ generation, economic growth, and government expenditure in education—underlying the transmission of educational advantage or disadvantage from one generation to the next.

We find that education mobility is not linear across the conditional distribution of the educational attainment of sons. For the overall sample and the subgroups, sons are most likely to move beyond their circumstances and not be dictated by their fathers’ educational status at the top tail of the sons’ conditional education distribution. The ‘*higher inequality leading to lower mobility*’ nexus in education plays out for the Indian scenario and thus corroborates the “Great Gatsby Curve.” Also, economic growth and public investment in education affect intergenerational education mobility positively.

For equality of opportunity to improve in society, public institutions need to play a significant role and devise policies to offset the disadvantage faced by the lowly endowed sections of the population. Given the high degree of education persistence at the primary and middle school levels across all subgroups, the government must follow the following directions. First, redistributive education policies should be designed to ensure primary and secondary education, irrespective of socio-economic status. Secondly, considering the spatial differences in mobility between urban and rural regions across the entire education distribution, it is essential to improve accessibility and the quality of education in rural areas of the country. Finally, enhancing access and upgrading the quality of higher educational institutions would go a long way in containing the wage premium and reducing the heterogeneity in returns to higher education in India, in turn suppressing the transmission of inequality and its effects.

The pronounced stratification in India’s education system, characterized by elite private schools, well-funded public schools, and under-resourced government schools, creates a tiered structure that impacts students’ educational and career outcomes differently. The vocational education system, perceived as a ‘dead-end’ track with limited upward mobility, adds another layer to this stratification (Mehrotra, 2014). Such an educational setting provides a rich context for exploring intergenerational educational persistence. Drèze and Sen (2013) and Desai and Kulkarni (2008) highlight significant disparities in educational quality and opportunities, exacerbated by a rigid tracking system post-grade 10 that further entrench inequalities. Future research should investigate how these systemic divisions impact long-term educational and career outcomes, aligning with de Werfhorst et al. (2010) and Pfeffer (2008), who emphasize the role of institutional structures in perpetuating educational inequalities.

Additionally, our study focuses on educational mobility, leaving other dimensions of socio-economic status unexplored. To address this limitation, future research could explore multifaceted aspects of intergenerational mobility. Longitudinal studies would strengthen causal inferences, while regional analyses could unveil variations influenced by evolving economic landscapes. Our findings contribute valuable stylistic insights, emphasizing the need for nuanced, evidence-based policies to ensure mobility and mitigate potential negative consequences, especially among diverse demographic subgroups in India.

## Data Availability

Publicly available datasets were analyzed in this study. This data can be found here: Indian Human Development Survey 2 (https://ihds.umd.edu/data/ihds-2).

## Footnotes

^1^English is a marker of economic and social status (Chattopadhay and Roy, 2017). The knowledge of English plays a key role in higher education as it is a widely used medium in higher education, and this promotes upward social mobility (Muralidharan and Kremer, 2008).
