# Supplementary material for: Intergenerational education mobility in India: nonlinearity and the Great Gatsby Curve
Source: Front Sociol. 2024 Sep 26;9:1295550. doi: 10.3389/fsoc.2024.1295550 (PMC11465184; doi:10.3389/fsoc.2024.1295550)
Supplement: Supplementary file 1 [file Table_1.docx]

Intergenerational Education Mobility in India: Nonlinearity and the Great Gatsby Curve

# Appendix

## **More Summary Statistics**

Table A1 reports the sample means of education attainment by age/birth cohorts. This data showcases that there has been a clear and steady growth in educational attainment over the years and across generations. Sons have consistently exceeded their fathers w.r.t. the time they have spent in school since India’s independence in 1947.

**Table A1. Summary Statistics by Age Cohorts**

| Son’s Age Cohort | Sample Size | Percent | Average Years of Schooling | |
| --- | --- | --- | --- | --- |
|  |  |  | **Son** | **Father** |
| 25-29 | 7,827 | 17.58 | 8.842 | 4.658 |
| 30-34 | 6,702 | 15.05 | 8.213 | 4.214 |
| 35-39 | 6,524 | 14.65 | 7.865 | 3.627 |
| 40-44 | 5,943 | 13.35 | 7.151 | 3.175 |
| 45-49 | 5,738 | 12.89 | 6.499 | 2.734 |
| 50-54 | 4,660 | 10.46 | 6.332 | 2.776 |
| 55-59 | 3,882 | 8.72 | 5.989 | 2.493 |
| 60-64 | 3,256 | 7.31 | 5.645 | 2.109 |
| Total | 44,532 | 100 |  |  |

**Notes:** The ages are as of the year 2011. Thus, the age cohorts could also be understood as the following birth cohorts – 1982-1986, 1977-1981, 1972-1976, 1967-1971, 1962-1966, 1957-1961, 1952-1956, and 1947-1951.

Next, we look at the rate increases in schooling attainments per annum. To obtain the same, we regress the number of years of schooling on the birth year of individuals (sons). The coefficient estimates (Table A2) mark the respective rates of schooling increases for the overall sample and various categorizations.

**Table A2. The average growth rate in educational attainment (Dependent Variable – ‘year of birth’)**

|  | Growth per Year | N |
| --- | --- | --- |
| Overall | 0.0910*** | 44532 |
|  | (0.00198) |  |
| Rural | 0.113*** | 28138 |
|  | (0.00234) |  |
| Urban | 0.0529*** | 16394 |
|  | (0.00321) |  |
| Brahmins & Other UCs | 0.0666*** | 13124 |
|  | (0.00359) |  |
| OBCs | 0.0953*** | 17981 |
|  | (0.00295) |  |
| SCs and STs | 0.126*** | 12702 |
|  | (0.00352) |  |
| Hindu | 0.0928*** | 36369 |
|  | (0.00218) |  |
| Muslim | 0.0933*** | 5264 |
|  | (0.00557) |  |
| Others | 0.0920*** | 2899 |
|  | (0.00725) |  |

**Notes:** Standard errors clustered at household level in parentheses; * p<0.05, ** p<0.01, *** p<0.001

From regression 1 of Table A2, the yearly increase in the educational attainment of sons in the sample is 0.09 years. This translates to an increase of approximately 0.9 years of schooling per decade. Convergence in educational attainment can also be observed between rural and urban India, as the growth in schooling outcomes in rural areas is almost twice that of urban regions. Similar convergence can also be discerned for the rates between upper castes and the rest.

## **Intergenerational Education Mobility: Bringing Mother and Daughters into Conversation**

In the overall sample spanning all age groups, fathers are co-residents with their respective sons or daughters in 44.08 percent of all cases, and mothers are co-residents with their respective offspring in 51.17 percent of the cases. We consider sons and daughters no longer enrolled in school (construed as having quit schooling or completed education) and their co-resident parents (mother and father) as our matched sample. As measures of parental education, we use the number of years of schooling of both the mother and the father of each individual. We estimate the following model using an ordinary least squares (OLS) estimation to compute the intergenerational regression coefficients (IGRCs) associated with mothers and fathers:

|  | $C_{i}=\beta_{0}+\beta_{1}F_{i}+\beta_{2}M_{i}+x_{i}^{'}\theta+\epsilon_{i}$ | (B-1) |
| --- | --- | --- |

Where, $C_{i}$ is the number of years of schooling of the child (son/daughter), and $F_{i}$ and $M_{i}$ are the number of years of schooling for the father and mother, respectively. The coefficients, $\beta_{1}$ and $\beta_{2}$, are the IGRCs associated with the father and mother, respectively. $x_{i}^{'}$ is the vector of control variables, including a categorical variable for whether the individual is a female and dummies for caste, religion, and state of the individual. Table B1 contains the regression results.

**Table B1. Intergenerational Regression Coefficients (All India) for co-resident children and their parents (Dependent Variable – Child’s YoS)**

|  | (1) | (2) | (3) | (4) | (5) | (6) | (7) |
| --- | --- | --- | --- | --- | --- | --- | --- |
|  | Overall Sample | | Caste | | | Religion | |
|  | (11-64) | (25-64) | Brahmins  and other  UCs (25-64) | OBCs  (25-64) | SCs &  STs  (25-64) | Hindu  (25-64) | Muslim  (25-64) |
| Father’s YoS | 0.346*** | 0.363*** | 0.300*** | 0.394*** | 0.391*** | 0.355*** | 0.395*** |
|  | (0.007) | (0.010) | (0.017) | (0.016) | (0.021) | (0.011) | (0.031) |
| Mother’s YoS | 0.219*** | 0.210*** | 0.216*** | 0.227*** | 0.214*** | 0.210*** | 0.260*** |
|  | (0.009) | (0.011) | (0.017) | (0.020) | (0.029) | (0.012) | (0.042) |
| Female (=1) | -0.77*** | -0.89*** | -0.47*** | -1.19*** | -1.07*** | -0.98*** | -1.15*** |
|  | (0.060) | (0.129) | (0.216) | (0.206) | (0.257) | (0.149) | (0.364) |
| Constant | 8.504*** | 8.591*** | 8.736*** | 7.951*** | 7.937*** | 7.495*** | 7.482*** |
|  | (0.267) | (0.304) | (0.344) | (0.641) | (0.637) | (0.363) | (0.403) |
| Caste Dummies | Yes | Yes | No | No | No | Yes | Yes |
| State Dummies | Yes | Yes | Yes | Yes | Yes | Yes | Yes |
| Religion Dummies | Yes | Yes | Yes | Yes | Yes | No | No |
| N | 24,090 | 11,529 | 3,927 | 4,681 | 1,769 | 9,308 | 1,386 |
| adj. R-sq | 0.350 | 0.335 | 0.329 | 0.324 | 0.277 | 0.334 | 0.319 |

**Notes:** Standard errors clustered at household level in parentheses; * p<0.05, ** p<0.01, *** p<0.001

In Table B1, the IGRCs for the overall sample and the sub-samples indicate a lower degree of intergenerational persistence (or a higher degree of intergenerational mobility) when compared to IGRCs in Tables 2, 3, and 4. However, this sample cannot be considered representative of the population due to the limitation imposed by the co-residency condition. The coefficients (IGRCs) reflect a downward truncation bias. It is not possible to estimate the magnitude of the truncation bias as the mother’s educational attainment cannot be ascertained for all the eligible individuals in the data, barring the co-resident combinations. Qualitatively, the patterns in these results (Table B1) are similar to those obtained from the representative father-son pairs in the primary sample (Tables 3, 4, and 5). The IGRCs in the columns labeled 1 and 2 in Table B1 for the overall sample show that children exhibit greater education mobility with reference to their mothers than their fathers. Moreover, within the caste-based subsamples, while Brahmins and other upper-cast individuals display higher mobility apropos of their father compared to OBCs and, SCs & STs, the same distinction cannot be made for education mobility comparisons between the upper-caste individuals and SCs & STs with reference to their mothers. Finally, w.r.t. mothers and fathers, Hindu individuals are more mobile than their Muslim counterparts.

## **The Great Gatsby Curve**

**Table C1.** **State-wise Gini Coefficients of Education and IGRCs**

| State | Education Gini | IGRC |
| --- | --- | --- |
| Daman & Diu | 0.334 | 0.49 |
| Meghalaya | 0.316 | 0.586 |
| Orissa | 0.315 | 0.538 |
| Arunachal Pradesh | 0.304 | 0.335 |
| Gujarat | 0.299 | 0.528 |
| Dadra & Nagar Haveli | 0.298 | 0.627 |
| Madhya Pradesh, Chhattisgarh | 0.297 | 0.495 |
| Karnataka | 0.295 | 0.469 |
| Maharashtra | 0.286 | 0.379 |
| West Bengal | 0.284 | 0.668 |
| Rajasthan | 0.270 | 0.537 |
| Tripura | 0.267 | 0.343 |
| Kerala | 0.264 | 0.352 |
| Andhra Pradesh | 0.259 | 0.55 |
| Tamil Nadu | 0.258 | 0.452 |
| Uttar Pradesh, Uttarakhand | 0.249 | 0.551 |
| Bihar, Jharkhand | 0.249 | 0.598 |
| Sikkim | 0.248 | 0.221 |
| Himachal Pradesh | 0.247 | 0.273 |
| Assam | 0.244 | 0.48 |
| Pondicherry | 0.228 | 0.718 |
| Mizoram | 0.223 | 0.454 |
| Delhi | 0.215 | 0.436 |
| Nagaland | 0.214 | 0.223 |
| Haryana | 0.213 | 0.38 |
| Punjab | 0.209 | 0.459 |
| Goa | 0.209 | 0.18 |
| Chandigarh | 0.195 | 0.423 |
| Jammu & Kashmir | 0.191 | 0.404 |
| Manipur | 0.158 | 0.441 |
